# Novel Treatments in Myasthenia Gravis

**DOI:** 10.3389/fneur.2020.00538

**Published:** 2020-06-30

**Authors:** Deepak Menon, Carolina Barnett, Vera Bril

**Affiliations:** Ellen & Martin Prosserman Centre for Neuromuscular Diseases, University Health Network, University of Toronto, Toronto, ON, Canada

**Keywords:** myasthenia gravis, treatment, immunotherapy, complement, Fc receptor

## Abstract

Myasthenia gravis (MG) is the prototypical autoimmune disorder caused by specific autoantibodies at the neuromuscular junction. Broad-based immunotherapies, such as corticosteroids, azathioprine, mycophenolate, tacrolimus, and cyclosporine, have been effective in controlling symptoms of myasthenia. While being effective in a majority of MG patients many of these immunosuppressive agents are associated with long-term side effects, often intolerable for patients, and take several months to be effective. With advances in translational research and drug development capabilities, more directed therapeutic agents that can alter the future of MG treatment have been developed. This review focuses on the aberrant immunological processes in MG, the novel agents that target them along with the clinical evidence for efficacy and safety. These agents include terminal complement C5 inhibitors, Fc receptor inhibitors, B cell depleting agents (anti CD 19 and 20 and B cell activating factor [BAFF)]inhibitors), proteosome inhibitors, T cells and cytokine based therapies (chimeric antigen receptor T [CART-T] cell therapy), autologous stem cell transplantation, and subcutaneous immunoglobulin (SCIG). Most of these new agents have advantages over conventional immunosuppressive treatment (IST) for MG therapy in terms of faster onset of action, favourable side effect profile and the potential for a sustained and long-term remission.

## Introduction

Myasthenia gravis (MG) is the prototypical autoimmune disorder caused by specific autoantibodies at the neuromuscular junction. Broad-based immunotherapies, such as corticosteroids, azathioprine, mycophenolate, tacrolimus, and cyclosporine, have been effective in controlling symptoms of myasthenia (1). Corticosteroids are effective in a majority of MG patients; however, these are associated with many long-term side effects, often intolerable for patients. Traditional steroid-sparing agents have shown mixed efficacy in trials, and usually take several months to be effective. Recently more directed, novel immunotherapies have been developed. These include terminal complement C5 inhibitors and Fc receptor inhibitors (2). These treatments work at different points of the immune pathology and are likely to be complementary in action. FC receptor inhibitors reduce the level of circulating pathogenic autoantibody, whereas terminal complement C5 inhibitors block the formation of the membrane attack complex at the last step of immune injury. This review discusses novel agents that act on other nodal points in MG pathogenesis, autologous stem cell, and chimeric antigen receptor T (CART-T) cell therapy in MG. These new treatments may help reduce the use of steroids, and their relatively fast onset of action makes them attractive options to traditional steroid-sparing agents. These treatments usher in a new era of more focused MG management that promises to improve the lives of people with MG.

## Overview of Myasthenia Gravis Pathogenesis

MG is an antibody mediated disease in which the immunopathogenesis is T cell driven and there exists a complex interplay between CD4+ T cells and B cells. The normal immune system weeds out autoreactive T cells early and these are destroyed in the thymus by the process called central tolerance. Autoreactive T cells that escape this process or arise *de novo*, are kept in check in the peripheral circulation by a subset of CD4+ cells called Treg cells that bring about apoptosis, anergy or suppression of autoreactive cells (3). These T reg cells which are outsourced from the thymus gland are crucial in maintaining immune tolerance and are found to be functionally deficient in MG. The immunological process in MG begins when immune tolerance is broken by a hitherto unidentified trigger, probably infectious agents, with “molecular mimicry” between the infectious antigen and the acetylcholine receptor(AChR) protein (4). The antigen presenting cells submit the AChR to the CD4+ cells leading to upregulation of proinflammatory cytokines such as interleukins and tumor necrosis factors (5). Also the defect in Treg cells results in upregulation of the proinflammatory CD4+ T cell effector subtypes Th1, Th2, Th17, and these stimulate B cells which proliferate to plasma blasts, plasma cells, and memory B cells (2, 6, 7). The antibodies secreted by the plasma cells in AChR antibody positive MG are mainly of IgG1 and IgG3 subclass (8, 9). These antibodies bring about the pathogenic immune cascade, binding by their fragment binding (Fab) site to the AChR and by the fragment crystallization (Fc) portion to the respective Fc receptors (FcR) expressed in all immunocytes (10). The various FcR subfamilies for IgG are either activating receptors (FcγRI, FcγRIIa/c, FcγRIII) or inhibitory receptors (FcγRIIb). Agents that modulate the function of these receptors are being recognized as novel therapeutic agents in many autoimmune diseases. While germinal centers in the thymus gland are the primary site of anti-AChR producing B cells, later, secondary lymphoid organs in the periphery can take over this function (11). Also, the integrity of the NMJ and effective AChR clustering depend on the effective interaction of other post-synaptic proteins such as muscle specific kinase (MuSK), low density lipoprotein receptor-related protein 4 (LRP4), agrin and rapsyn, amongst others. The binding of agrin to LRP4 results in dimerization of MuSK which is vital for effective AChR clustering in the post-synaptic membrane (5, 12). Antibodies against MuSK and LRP4 have been found to be pathogenic in MG. The pathogenic process is different in muscle specific kinase (MUSK) MG in that the thymus is not involved in the pathology and the MuSK antibody, belonging to IgG4 subclass, does not activate the complement system. This binding of anti-MuSK antibodies masks the site for normal MuSK-LRP4 interaction, thus preventing acetylcholine receptor clustering necessary for normal neuromuscular function (5). The anti-LRP4 antibodies belong to the IgG1 subclass, and, in addition to disrupting LRP4-agrin interactions, also activate the complement pathway leading to damage of the NMJ (12). Knowledge of the various processes involved in the immunopathogenesis of MG has led to identification of potential targets that can selectively inhibit the immune cascade leading to MG.

## Conventional Treatment of Myasthenia Gravis

**Figure 1:**
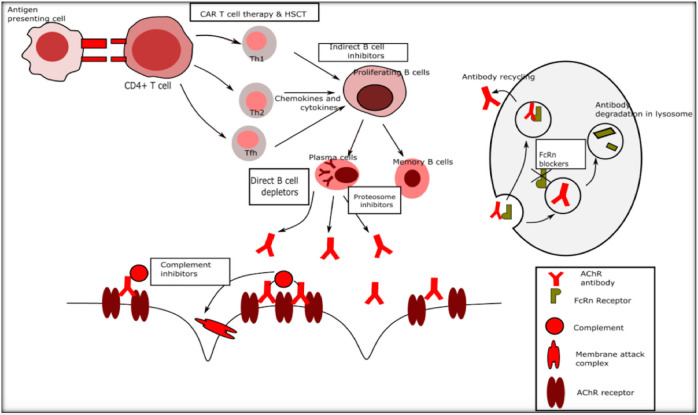
Immune targets for novel therapies in myasthenia gravis. Th1 Type 1 T helper cells, Th2 Type 2 T helper cells.

The existing standard of care in the management of myasthenia gravis includes ‘broad-spectrum' immunosuppressive treatment (IST) with medications such as corticosteroids, azathioprine, mycophenolate, methotrexate, cyclosporine, tacrolimus, and immunomodulatory treatments such as plasma exchange (PLEX) and intravenous immunoglobulin (IVIG) (1). The mechanisms of action of immunosuppressive agents include activating or suppressing target genes and thereby causing a multitude of changes including suppression of antigen production and reducing circulating T cells (corticosteroids), interfering with T and B cell proliferation by cell cycle arrest (azathioprine, methotrexate, and mycophenolate), inhibition of T cell activation (cyclosporine, tacrolimus), inhibition of antigen presenting cell interaction with T cells and Fc receptor blockade among other actions (IVIG) (13–15). While these treatments are time-tested and remain the commonly used agents for MG, the disadvantages are many such as increased susceptibility to life threatening infections, a wide range of deleterious systemic side effects, delayed onset of action and minimal but definite increased long term risk of malignancy and drug toxicity. With advances in immunology, molecular biology and drug development, newer agents that have more selective immunological targets, spare the rest of the immune system, with lesser toxicity, and more rapid onset of action with possibly sustained remission and cure, are being developed at a rapid pace. Figure 1 outlines the immune system and potential targets for novel therapies.

## Novel Immunotherapies for MG

### Complement Inhibitors in MG

Once the binding of IgG to the AChR epitopes has occurred, it sets in motion the cascades of the classical and common complement pathways. The final steps in the cascade result in formation of C5 convertase, which splits C5 into C5a and C5b. C5b combines with C6-C9 factors to form the membrane attack complex (MAC) which incorporates into the cell membrane resulting in cell damage and lysis (16, 17). This is evidenced by the presence of IgG, C3, and MAC deposits at the neuromuscular junction in affected postsynaptic membrane and also by the low circulating complement levels due to the consumption of these factors, both in affected humans and in animal models (18). The low complement titres correlate with clinical severity and with higher levels of AChR antibodies (19). Moreover, complement knockout mice have significantly lower incidence and severity of experimental autoimmune myasthenia gravis (EAMG) (20). These observations led to studies of several complement inhibitors such as cobra venom factor, soluble complement receptor 1, anti C5 and anti C6 antibodies. All showed improvement in AChR content at the post synaptic junction, reduced MAC deposition despite elevated levels of IgG, and a parallel improvement in muscle weakness in animal models (18, 21). Based on this evidence, anti C5 antibodies that inhibit the common complement pathway were developed, but had the disadvantage of increased risk of opportunistic infections leading to further development of molecules such as small interfering RNAs that selectively inhibit the classical complement pathway, the latter being in preclinical development (22). The role of the complement system in seronegative MG (SNMG), and therefore the utility of complement inhibitor therapy in this group of patients, is questionable. However, recent pathological evidence based on samples from external intercostal muscle biopsies from patients with SNMG showed complement deposition at the NMJ, suggesting importance of complement in this subgroup of patients with MG (23). The efficacy of complement inhibitors in SNMG remains to be demonstrated in appropriate clinical trials.

### Eculizumab

Eculizumab is a recombinant humanized monoclonal antibody that binds to C5 complement and prevents its cleavage to active C5a and C5b factors and is the first available drug that targets the complement system, specifically C5 (24). It was initially approved by the Food and Drug Administration (FDA) for the treatment of paroxysmal nocturnal hemoglobinuria in 2007 and has since then been approved for use in atypical haemolytic uraemia, generalised MG and neuromyelitis optica (Table 1) (40, 41).

**Table 1:** Novel immune therapies for treatment of myasthenia gravis.

**Agent**	**Action**	**FDA approval/Ongoing trials**	**MG serology and type**	**Route**	**Dosage and interval**	**Main safety concerns**	**Remarks**
Eculizumab	C5 inhibitor	FDA approved	AChR positive gMG	IV	900 mg weekly x 4 weeks, followed by 1,200 mg every alternate weeks	Neisseria meningitis, infections	Vaccinate at least 2 weeks prior (25)
Zilucoplan	C5 inhibitor	Ongoing phase III trial(RAISE) in MG, (26) FDA orphan drug approval	AChR positive gMG	SC	0.3 mg/kg daily	Concerns of meningitis	
Ravulizumab	High affinity C5 inhibitor	Ongoing phase III trial in MG (27)	Unspecified gMG	IV	Weight based 2400–3000 mg every 15 days	Headache	FDA approved for PNH,
Efgartigimod	FcRn blocker	Ongoing phase III trial (28)	AChR positive gMG	IV	10 mg/kg weekly	Headache, reduced monocyte count	
Nipocalimab	High affinity FcRn blocker	Ongoing phase II (29)	Unspecified gMG	IV	Every 2 weeks (multiple doses, under phase II study)		Potentially safe in pregnancy
Rozanolixizumab	High affinity FcRn blocker	Ongoing phase II (30)	AChr or MuSK positive gMG	SC	7 mg/kg once a week	Headache forcing withdrawal,	No increased infection in trials
RVT 1401	FcRn blocker	Ongoing phase II (31)	AChR positive	SC or IV	340/680 mg weekly for 4 weeks followed by 340 mg every 2 weeks	No severe adverse effects	
Rituximab	Anti CD20 antibody	Phase II trial, data unpublished	AChR or MuSK positive gMG	IV	375 mg/m^2^ body surface area per week for 4 weeks, repeated after 6 months	Infusion reactions, rare long term risk of PML	Second line option especially in refractor MUSK positive MG
Belimumab	BAFF inhibitor	Phase II trial, no significant benefit to standard of care (32)	AChR or MuSK positive	IV	10 mg/kg at 2–4 weeks interval	Influenza, gastric side effects	No ongoing trials
Bortezomib	Proteosome inhibitor	Phase II trial terminated due to recruitment issues (33)	AChR positive, anecdotal reports in MuSK positive	SC	2 cycles, each consisting of 2 doses of 1.3 mg/m^2^ body surface area, at 10 day intervals	Sensory motor polyneuropathy	May require acyclovir and trimethoprim-sulfamethoxazol prophylaxis
CAR T cell therapy	Autologous T cell directed against BCMA	Ongoing phase I and phase II trials (34)	Not specified gMG	IV		Cytokine release syndrome	FDA approved for refractor B cell leukemia and lymphoma
Hematopoetic stem cell transplantation	Ablation of auto-reactive T and Memory B cells	Ongoing Phase II (35)	Ideally in seropositive MG	IV		Complications related to conditioning regime	
SCIG	Broad spectrum immunomodulation	Phase II trials, As efficacious as IVIG, better patient satisfaction (36–38)	AChR or MuSK positive	SC	IVIG equivalent dose weekly divided dose	Injection site reactions	For maintenance treatment
Monarsen	Antisense oligonucleotide against ACHE-R isoform	Phase II trial (2008), Modest improvement (39)	AChR positive	Oral	500 mg/kg	None	No ongoing trials

A large phase 3 trial (REGAIN) showed major benefits in patients with refractory generalized MG although the response rate was not 100% and most patients required ongoing chronic therapy with other ISTs. The REGAIN study enrolled 125 patients with refractory generalised MG randomized to either intravenous eculizumab or placebo as an add on medication to existing IST treatments, excepting PLEX and IVIG, for 26 weeks,. The dosage schedule was induction with 900 mg on days 0, 7, 14, and 21; 1200 mg at week 4; and then maintenance dosing of 1,200 mg every second week for 26 weeks. The primary efficacy endpoint was the change from baseline to week 26 in total score of MG-Activities of Daily Living (MG-ADL) measured using worst-rank ANCOVA. The trial did not meet the primary outcome efficacy parameter, but multiple secondary end-point measures such as change in MG-ADL, Quantitative MG (QMG) score and MG-Quality of Life (MG-QOL15 scores) showed significant improvement from baseline values in the eculizumab group compared with placebo (42). Eculizumab inhibits complement at the last stage of the immune cycle as noted above, but does not change abnormal antibody production and other potential immune mechanisms underlying MG. The ongoing requirement for other ISTs as observed in the open-label extension study is likely due to the presence of these other immune mechanisms, such as blocking or cross-linking effects of the abnormal antibodies that are unaffected by eculizumab. A major potential risk of eculizumab is that of meningococcal meningitis leading to the need for appropriate immunization prior to the initiation of eculizumab therapy. Hence Neisseria meningitidis vaccination is advised at least 2 weeks before starting treatment and revaccination after 2–5 years (25). If vaccination is not possible before beginning treatment, then prophylactic antibiotics are advised until 2 weeks after vaccination.

The results of the REGAIN trial led to approval for the use of eculizumab in refractory generalized, AChR antibody (AChRAb) positive MG by the FDA, Health Canada, the European Medicines Agency (EMA) and the Pharmaceuticals and Medical Devices Agency (PMDA) in the US, Canada, Europe and Japan respectively. In the open label extension of REGAIN where 117 patients received 1,200 mg every 2 weeks for a median of 22.7 months, there was 1 case of meningococcal meningitis which resolved with antibiotics. Infections occurred in about 19% of patients including infections with pseudomonas, cytomegalovirus and aspergillus as well as septic shock. A significant reduction in exacerbation rates, MG related hospitalization and rate of rescue therapy was seen in the double blind study and most patients reported global clinical improvement. More than half of the patients achieved minimal manifestation status or pharmacological remission (43). Additional analyses of the data from the REGAIN and the open-label extension studies have confirmed the benefits of eculizumab treatment as more refractory MG patients on eculizumab have minimal symptom expression compared with those on placebo (44). The success of eculizumab has led to the development of other complement inhibitors.

### Zilucoplan

Working in a similar fashion to eculizumab, zilucoplan is a synthetic macrolide peptide complement inhibitor that prevents cleavage of C5 complement protein into active C5a and C5b fragments, thus preventing downstream formation of MAC (45). A recent phase II randomized placebo-controlled trial compared two doses of subcutaneous zilucoplan in patients with moderate to severe generalised MG (defined as QMG score ≥ 12), and positive AChR antibodies. The higher dose group (0.3 mg/kg daily) achieved significantly lower mean QMG and MG ADL scores (primary end points) and also lower MG composite (MGC) and better MGQOL (secondary end points) at 12 weeks compared to baseline and no patient required rescue therapy. There were no serious side effects reported and minor side effects included injection site reactions (46). A phase III study is currently under way to study the safety, efficacy and tolerability of zilucoplan in AChRab positive patients with moderate to severe generalised MG (26).

### Ravulizumab

Ravulizumab, another humanized monoclonal antibody, is a novel C5 complement inhibitor which differs from eculizumab by aminoacid substitutions in the Fc region of eculizumab that provide a high affinity for C5 and immediate and sustained reduction in C5 (47). This modification confers a longer half life of the antibody due to recycling through the FcRn pathway. As a result, patients can be given ravulizumab every 8 weeks, an advantage over eculizumab which is administered biweekly. Phase 3 trials have shown the outcome of ravulizamb to be non-inferior to eculizumab in PNH and this medication has been approved by the FDA for the treatment of PNH (48, 49). A phase III randomized placebo-controlled multicentre study to evaluate the safety and efficacy of ravulizumab administered once every 15 days in generalized MG is underway. The serological status was not specified (27).

### Fc Receptor Inhibitors

Among the Fc receptors, neonatal FcR (FcRn) play a pivotal role in maintaining IgG homeostasis and are recognized as a treatment target in myasthenia. While initially recognized as the mediators of passive transfer of immunity from mother to fetus, their role in protecting IgG from lysosomal degradation and prolonging the half-life of immunoglobulins, has been recognized subsequently (50). By blocking the FcRn receptor, the recycling of IgG is reduced because IgG is being degraded in lysosomes. Since production of IgG does not compensate for this decrease, FcRn receptor blockade causes a rapid fall in all IgG subclasses (50, 51). In rat models of MG, treatment with anti-FcRn-antibody showed significant reduction in severity of symptoms and lowering of total and anti-AChR IgG levels providing pre-clinical proof of concept (52).

### Efgartigimod

Efgartigimod is a mutated human IgG1 Fc portion with increased affinity for FcRn at both physiological and acidic pH, whereas regular IgG-FcRn binding occurs strictly in acidic pH (53). In healthy volunteers, a single dose of 50 mg/kg reduced the total IgG by about 50% and multiple doses further reduced IgG levels by a total of 75%, with return to near baseline levels after ~8 weeks (54). The study subjects had only minor adverse effects such as headache and chills at higher doses. In a phase II study of 24 patients with generalized, AChRab positive MG, on stable doses of standard treatment, randomized to IV efgartigimod (maximum dose of 1200 mg per infusion) or placebo for 3 weeks showed safety and tolerability of efgartigimod. The most common adverse events were headache and reduced monocyte counts which were minor. A rapid reduction in all IgG subclass levels was observed in the first week after treatment, and further decreases to a total 70% reduction from baseline levels with subsequent doses. There was a gradual but incomplete return to baseline levels (20% reduction) at 8 weeks after treatment, with parallel changes in AChRab levels. Interestingly, there were improvements in all the MG scales used in the study, and these mirrored the fall in IgG levels, but persisted even after the IgG levels had increased close to baseline levels (55). A phase III study is currently underway to evaluate the efficacy and safety of efgartigimod 10 mg/kg per week for 26 weeks in patients with generalised AChRab positive MG (28).

### Nipocalimab

Nipocalimab (M281) is a fully humanized monoclonal IgG1 anti-FcRn antibody which binds with extremely high affinity to FcRn both at endosomal and extracellular pH blocking the binding of IgG to FcRn. It occupies the FcRn receptor throughout the cell cycle and has high specificity, minimizing off-target effects, and is unlikely to cross the placenta (53). A phase I placebo controlled study in 50 subjects examined both single (at 0.3, 3, 10, 30, and 60 mg/kg) and multiple ascending doses (four weekly doses of 15 or 30 mg/kg). Nipocalimab achieved rapid FcRn receptor occupancy and up to 80% reduction in IgG levels with 30 or 60 mg/kg doses and 50% reduction persisting for 18 and 27 days respectively for 30 or 60 mg/kg doses. There were no severe or serious adverse effects or increased risk of infections (56). A phase II trial is underway in AChRab or MuSK positive generalised MG exploring the safety and efficacy of nipocalimumab (29). An added advantage is its probable safety profile in pregnant women, and a clinical trial is now underway in pregnant women at high risk for severe haemolytic disease of the fetus and newborn (57). Because women of reproductive age constitute a large proportion of early-onset MG cases (58), interventions compatible with pregnancy are much needed.

### Rozanolixizumab

Rozanolixizumab is a humanized high affinity anti FcRn monoclonal IgG4 antibody. A four week study in cynomologus monkeys showed marked decrease (75–90%) in IgG concentrations with 50 and 150 mg/kg doses every 3 days for 4 weeks, with maximum effect by day 10. There were no safety concerns or increased infections (59). In a phase I randomized placebo controlled study to evaluate safety, healthy subjects were randomized to single infusion of intravenous or subcutaneous doses of 1, 4, or 7 mg/kg of razonolixizumab. The most common adverse events were headache (38.9%), vomiting (25%), nausea (19.4%), and pyrexia (19.4%), all occurring more frequently with intravenous administration compared to subcutaneous treatment. The reduction in IgG concentration peaked at 7–10 days and gradually returned to baseline by day 57 (59). In a subsequent phase II trial, 43 patients with AChR or MuSK positive generalised MG were randomized to 3 weekly subcutaneous infusions of placebo or rozanolixizumab, and then 4 weeks later, were re-randomized to 3 weekly doses of either 4 or 7 mg/kg. Standard of care MG treatments were stable during the study. The study showed clinical benefits across several endpoints, including QMG, MGC and MG-ADL scores as well as marked reduction of total IgG and AChRab levels. There was a greater frequency of headache (57.1%) compared to placebo (13.6%) and three patients withdrew from the study due to headache (53, 60). A 240 patient, phase 3, parallel design, randomized, double-blind, placebo-controlled, multi-centre clinical study of rozanolixizumab is ongoing currently (30).

### RVT 1401

RVT 1401 is a fully humanized monoclonal FcRn antibody for subcutaneous or intravenous injection. Limited information from an unpublished phase 1 trial on healthy volunteers report that a single, subcutaneous, 765 mg dose of RVT 1401 reduced IgG by 47% with further reduction after continued weekly injections. All adverse events were mild to moderate in severity, with no subjects requiring premature discontinuation due to AEs (61). A phase II trial comparing weekly subcutaneous 680 and 340 mg RVT 1401 doses to placebo in patients with AChR antibody positive MG is in progress. The study also has an open label extension arm with 340 mg every 2 weeks for 6 weeks (31).

These studies of complement inhibitors and FcRn inhibitors are not without certain limitations. None of these trials have included seronegative MG patients though this group of patients may resemble antibody positive patients in response to immune therapies (62, 63). Longer treatment durations are necessary to confirm the long term efficacy and potential adverse effect of these agents. Since the patients in these trials were continued on stable doses of standard agents, the utility of these newer agents in crisis and the timing of their introduction into the care regimen remain uncertain. FcRn inhibition also has the potential to alter serum levels of therapeutic monoclonal antibodies and the pharmacokinetic interactions among these agents remain unexplored (64).

### B Cell Depleting Agents

As MG is primarily mediated by humoral mechanism, B cells play a central role in MG pathology.

### Role of B Cells in MG

Under the influence of the Tfh subset of CD4 T cells and with regulatory Tfr CD4 T cells being defective, B cells differentiate into memory B cells, plasmablasts and plasma cells in the thymic germinal centers (65). The plasma cells are the terminal differentiated effector B cells and along with plasmablasts secrete the pathogenic antibodies. The various plasma cell populations differ in their phenotypes in the expression of cell surface molecules, for example, CD20 which are less expressed in fully mature plasma cells and memory cells (66). In addition to their main pathogenic role in autoantibody generation, B cells also serve as efficient antigen-presenting cells to T cells and, by this means, trigger there activation and proinflammatory upregulation (67). The germinal centres of the thymus provide an ideal environment for differentiation and proliferation for autoreactive B cells (11). There are a number of molecular and cellular factors that influence this proliferation among which BAFF deserves special mention. Both normal and more importantly autoreactive B cells are very much dependent on BAFF for their survival and maturation (68). The beneficial effect of thymectomy is explained by the removal of the thymus associated germinal centers (69). However, after thymectomy the antibody levels do not disappear completely disappear from the serum. This may due to persisting memory B cells and long lived plasma cells which can be localized in secondary lymphoid organs and can replenish short lived plasma cells that secrete antibodies. The concept that long-lived plasma cells are not affected by IST drugs such as corticosteroids or cyclophosphamide, or by B cell depletion, has identified them as a novel target cell requiring specific therapeutic approaches (66). There are various steps at which B cells can be targeted either directly or indirectly.

## Direct B Cell Depletors

### Rituximab (RTX)

RTX has gained popularity in recent times and has been employed for MG in many centers across the world. This is despite that most of the data for RTX in myasthenia comes from single centre experiences and case series and its use remains an off-label treatment for myasthenia.

RTX was developed in the 2000s for cancer and other autoimmune disorders and is a murine-human chimeric anti-CD20 glycoprotein monoclonal antibody. CD20 is a transmembrane protein expressed by B cells, but not by long-lived plasma cells and plasmablasts. It can induce killing of CD20+ cells via multiple mechanisms. The direct effects of RTX include complement-mediated cytotoxicity and antibody-dependent cell-mediated cytotoxicity, and the indirect effects include structural changes, apoptosis, and sensitization of cancer cells to chemotherapy. RTX also increases Treg cells which favourably influences MG immunology. A systematic review of the efficacy and safety of RTX in MG (99 patients AChRab positive, 57 patients MuSK positive) showed that MG Foundation of America (MGFA) minimal manifestation status, or better, was achieved in 44% of patients, and combined pharmacological and complete medical remission was observed in 27%. MuSK positive patients had better response than AChRAb positive patients, with 72% of MuSK patients achieving minimal manifestation status or remission, compared to 30% of AChR positive patients. Relapses were also less frequent in MuSK MG. Other predictors of a positive response were younger age of onset and milder disease. Reduction in antibody titers was not correlated with clinical response to RTX in AChR or MuSK patients (70). A multi-centre, retrospective study of RTX in MuSK positive MG, also showed that individuals who received RTX had better outcomes than those on standard treatments (71). A recent, retrospective nationwide study from Austria showed that, at a median follow-up of 20 months, MG patients (70% AChRab positive, 25% MuSK positive) treated with rituximab achieved remission in about 43% and minimal manifestations in 25% (70). Remission was more frequent in MuSK positive patients than in AChRab positive patients (71 vs. 36%) (72). Another recent retrospective review from Stockholm showed that rituximab shortened the time to remission and the need for additional immunosuppressive therapies in patients with new-onset MG treated within 12 months after diagnosis, compared to the longer time for remission in those with refractory disease (73). The time to remission was shorter with rituximab compared to those who received other immunosuppressive therapy. A recent randomized controlled trial, compared RTX to placebo as add-on treatment in patients with AChRAb positive MG (the BEAT-MG study) (74). Patients were required to be on prednisone ≥ 15 mg/day with or without additional ISTs, and they received RTX or placebo every 6 months for 2 cycles, with final follow up at 52 weeks. At the end of the study, RTX did not have a corticosteroid-sparing effect compared to placebo; additionally there were no significant differences in outcomes of disease severity. However, the baseline scores on different outcome measures were relatively low, so it is possible that the population selected was too mildly affected to show significant change (75). While the full BEAT-MG results are currently unpublished, there may be other reasons for the negative results such as the potential development of human antichimeric antibody (HACA) against RTX. Also, since long lived plasma cells lacking CD20 are not targeted by RTX, any clinical benefits may be transient and would require chronic infusions—beyond the 2 cycles in the study— to maintain the effects (76).

Despite the lack of robust evidence, RTX is the second-line drug for treatment of MG in some areas of the world (2). The evidence for efficacy in MuSK MG is more robust, although randomized controlled trials are lacking. Since patients with MuSK MG tend to have refractory disease RTX has been proposed as first line of treatment in this population (2, 71). Although RTX may be safe for long-term use in MG, there is a risk, although low, of progressive multifocal leukoencephalopathy with this treatment, so its use needs to be cautious (77).

### Next Generation Anti-CD-19 and Anti-CD-20 Biologicals

Next generation anti-CD20 and anti-CD19 biologicals have been considered as possible treatments for MG. Most of the second generation anti-CD20 agents such as ocrelizumab, ofatumumab, obinutuzumab, veltuzumab, and ofatumumab have the advantage of being fully-humanized and thus may be better tolerated and more efficacious in haematological malignancies and autoimmune disorders (78, 79). Ofatumumab showed sustained remission in a patient with refractory MG who had previously responded to RTX but developed hypersensitivity reactions to repeated RTX infusion (80). Given the lack of phase III studies, there is insufficient data to recommend these newer agents for use in MG at present.

Anti CD19 agents offer several advantages over anti CD20 agents. CD19 is a B cell marker that is expressed much earlier than CD20 and, as a result, may be a better target and might possibly act synergistically with anti CD20 agents. The most promising anti CD19 agents are blinatumomab, SAR3419 and MEDI-551 which are currently in phase II studies in haematological malignancies (79).

## Indirect B Cell Inhibitors

### Belimumab

Belimumab (Benlysta, Rockville, MD), is a human immunoglobulin (Ig) G1λ monoclonal antibody against B-lymphocyte stimulator (BLyS) also called BAFF. Elevated BAFF levels have been identified in patients with MG, highlighting it as a potential treatment target (81).

BAFF belongs to the tumor necrosis factor (TNF) superfamily and is a costimulator for B-cell survival and function. The binding of BAFF to B cell receptor promotes the survival of the autoantibody-producing B cells by preventing their apoptosis. Transgenic mice overexpressing BAFF have excessive numbers of mature B cells and autoantibodies as well as an overall increased autoimmune response while BAFF deficient animals have marked reduction in B cells and hypogammaglobulinemia (82, 83). Belimumab has been approved by the FDA for the treatment of lupus (84). However, in a phase II randomized, placebo controlled trial in AChRab positive generalised MG, patients on belimumab did not have a significant difference in QMG scores or MG-ADL at week 24 compared to patients who were on placebo (32). While this might be due to a lack of effect of belimumab in MG, other potential reasons for the negative results include: a population of stable patients with mild disease, leading to floor effect of the MG scales, and exclusion of MuSK positive patients.

### Proteosome Inhibitors

The immune system contains long-lived memory plasma cells which are terminally differentiated B cells that have lost cell surface markers and are as a result resistant to most agents such as RTX. These plasma cells reside in niches and form sentinels of adaptive immunity (85). Such plasma cells have not been targeted and may be responsible for treatment resistance in autoimmune diseases. Given the high rate of immunoglobulin synthesis, these plasma cells are sites of high protein turn over. Many of these cellular proteins need effective degradation and removal for cellular homeostasis. Proteosomes are hollow, cylindrical protein structures which are an integral part of the ubiquitin-proteosome pathway that plays a major role in clearing intracellular proteins (86). Inhibition of proteosomes causes accumulation of misfolded proteins and apoptosis of highly active plasma cells and this therapy is employed in treatment of multiple myeloma (87). In EAMG animals, bortezomib efficiently reduced the rise of AChRab titers, prevented ultrastructural damage of the postsynaptic membrane, improved neuromuscular transmission, and decreased myasthenic symptoms (88). An open label trial to investigate the use of bortezomib in treatment of resistant autoimmune diseases including MG, SLE and RA was terminated early due to recruitment issues (33, 89). Bortezomib was tried in a patient with resistant MuSK positive MG with moderate improvement, but the patient had received RTX nineteen days before the initiation of bortezomib, a major confounder (90). Although bortezomib may be promising in MG, further studies are needed. A limiting factor is the potential for development of sensory neuropathy observed in 30–40% of those treated with bortezomib, and this neuropathy is disabling and permanent in some patients (87). More selective inhibition of the proteosome subunit, called the immuneproteosome which may have less neurotoxicity, is in preclinical development. ONX 0914, an immuneproteosome inhibitor, reduced the severity of EAMG through varied mechanisms including reduction of autoantibody affinity, and reduction of Tfh cells and antigen presenting cells, but additional studies are required prior to clinical use (91).

### T Cells and Cytokine Based Treatment in Myasthenia

With Treg cell dysfunction and Th1, Th2, and Tfh over action being major factors in MG pathogenesis, agents that target T cells, promoting regulation or inhibition, may be attractive options for MG treatment. Given that Th1, Th2, and Tfh cells act through various cytokines to induce B cell proliferation and differentiation into plasma cells, drugs designed to inhibit cytokines are also attractive treatment options (21). Animal models with inborn deficiencies of cytokines and those treated with cytokine inhibitors of IL1, IL6, and TNF, were resistant to EAMG (22, 92, 93). Several monoclonal antibodies have been developed to target Th cells or cytokine pathways. These include secukinumab (inhibits IL 17A), rontalizumab (inhibits INFalpha pathway), and tocilizumab (inhibits IL6 pathway) (6). Many of these agents have been approved for treatment of psoriasis and psoriatic arthritis (6). Tocilizumab has been reported to be beneficial in patients with refractory MG, one of whom failed to benefit with RTX (94). At present, none of these agents are being studied in MG clinical trials.

## Chimeric Antigen Receptor—T (Car-T) Cell Therapy

The concept of adaptive T cell immunity had been evolving in cancer therapy, The concept is to treat patients with advanced cancer using their own T cells which have been harvested, manipulated *ex-vivo*, expanded and then re-infused. The presumed, increased effectiveness of a patient's own T cells against the malignancy is thought to occur by redirecting the native T cells against selected antigens expressed only by the tumor cells. The CAR-T cells are genetically engineered and expanded autologous T cells that are infused into the patient and recognize tumor cell antigens, and thus bring about tumor cell destruction (95). The major adverse effect of this therapy is the cytokine releasing syndrome (CRS) which can range from mild constitutional symptoms to severe CRS leading to multi-organ dysfunction (96). CAR-T cell therapy has received FDA approval for the treatment of refractory B cell acute lymphocytic leukemia, B cell lymphoma, and non-Hodgkin lymphoma, but is likely to have wider application in hemato-oncology (97). Applying these principles to treatment of autoimmune disorders, chimeric autoantibody receptor T (CAAR-T) cells have been developed to target autoreactive B cells secreting autoantibodies. Pre-clinical studies have found efficacy in various animal models of autoimmune disorders including autoimmune encephalomyelitis, lupus and pemphigus (98). Thus CAR T cell therapy offers a novel and attractive treatment opportunity in MG. Currently phase I and phase II trials are underway using CD8 positive CAR T therapy directed against plasma cells that express B-cell maturation antigen (BCMA) (34).

## Hematopoetic Stem Cell Transplant (Hsct)

The data for the use of HSCT in various refractory immune mediated neurological disorders have been accumulating over the past two decades, most notably for multiple sclerosis (99, 100). Autologous stem cell transplantation has the advantage over allogenic transplantation in having lesser risk for graft vs. host disease. The basic mechanism of action of HSCT is ablation of all existing autoreactive T cells and B cells, including memory cells and long-living plasma cells, during the conditioning phase using cytotoxic therapies or radiation, depending on the conditioning regime (101). The subsequent autologous hematopoetic transplantation helps in recovery from the post-conditioning aplasia and enhances immunotolerance by increasing regulatory T cells, reducing autoantibodies and rejuvenating thymic function (102, 103).

A retrospective case series of seven patients with severe refractory MG treated with HSCT showed that all patients were in complete stable remission at the median follow-up time of 40 months. At 8 months after HSCT, all patients had discontinued ISTs (104). An intensive conditioning regimen was employed in all patients but acute complications were transient and none of the patients required ICU care. A subsequent systematic review of HSCT therapy showed that 2.2% of all articles were in MG, 29.4% in graft versus host disease and 19.8% in multiple sclerosis (105). With better and safer induction regimens, HSCT may be a reasonable treatment option in severe refractory MG in the future. However, factors to consider in assessing these reports are whether these patients had received adequate trials with other immunosuppressants prior to transplant, and whether using only high dose cyclophosphamide induction, without transplant, would have induced sustained remission (106). A phase II trial to evaluate the safety and efficacy of high dose chemotherapy in autoimmune neurological disorders, including MG, is in progress (35).

## Subcutaneous Immunoglobulin (SCIG)

IVIG is a useful treatment option when a rapid response is required in worsening or poorly controlled MG. While there is class I evidence for the short term use of IVIG in acute worsening or myasthenic crisis, data for maintenance therapy is less robust, and is restricted to class III evidence (107–109). Immunoglobulins(Ig) have broad-spectrum immunomodulatory actions and exert their influence by a number of B-cell, T-cell, complement and Fc receptor modifying actions (110). However, some of these same actions and other factors such as increased blood viscosity, rapid exposure to high foreign protein load and rapid intravenous volume expansion lead to the frequent adverse effects of IVIG ranging from 2.5 to 87.5% with repeated infusions (111). Subcutaneously administered immunoglobulin (SCIG) has advantages over IVIG since the slow and sustained intravascular absorption avoids the abrupt vascular volume load, and subcutaneous administration eliminates the need for intravascular access. Many patients report improved quality of life (QOL) with greater freedom, control, and independence in their treatment with immunoglobulin (112). With these attractive advantages, SCIG was used initially in primary immunodeficiency disorders and was as effective as IVIG in preventing infections with a lower incidence of serious adverse events (113). The utility of SCIG as maintenance therapy for MG was examined in a retrospective case series of 9 patients. At a mean follow-up period of about 7 months, all had stable or improved MGFA status, significant improvement in MG-ADL, MG-QOL and the visual analogue scale (VAS) for patient satisfaction (36). The efficacy, safety and tolerability of SCIG in 22 seropositive MG patients was assessed in a multicentre North American open label trial (37). After a 10 week screening period with periodic IVIG treatments, stable patients were transitioned to weekly SCIG for 12 weeks. The study showed improved scores in the QMG, manual muscle testing (MMT), and MGC with high patient satisfaction and no serious adverse effects. The treatment success rate at 12 weeks was 85% (37, 38). Thus SCIG offers a novel, efficacious and patient-friendly alternative to IVIG in maintenance therapy for MG, although it has not been tested for acute management of MG. Additionally, its corticosteroid-sparing effects have not been established.

## Other Nonimmune Treatments

### Antisense Oligonucleotide Treatment Against Acetylcholinesterase

While acetylcholinesterase inhibitors (ACHEI) were the first agents to be tried in MG and provided symptomatic improvement, the focus of attention has shifted mainly to treating the primary aberrant immunological processes of MG. However, the use of an antisense oligonucleotide which hybridizes with ACHE mRNA may be a therapeutic option. Splicing of the ACHE gene normally produces different ACHE isoforms, the predominant one being the ACHE-S isoform in physiological condition. Acute exposure to anticholinesterases shifts the splicing of the AChE pre-mRNA to the normally rare, AChE-R variant (114). The increase in AChE-R levels enhances ACh hydrolysis and restores the balance between the ACh and AChE levels. The antisense oligonucleotide EN101, or Monarsen, targets exon 2 of the AChE mRNA and results in AChE-R mRNA being more susceptible to destruction which decreases its activity, and hence maintains levels of acetylcholine in the synaptic cleft (115). Monarsen, intravenous and oral, reduces AChE-R levels in EAMG rat muscle and plasma and enhances task performance. Initial phase 2a studies in MG patients showed modest improvement in QMG scores and that the treatment was safe and well tolerated (39). Additional studies of Monarsen are not underway at this time.

## Conclusion

The availability of more focused immune therapies provides greater treatment options for both patients and treating physicians in the management of MG. A favourable benefit-side effect profile and more rapid onset of action are advantages over current ISTs. However, the long term efficacy and safety of novel treatments are yet to be understood fully. Furthermore, the high and sometimes prohibitive cost of many novel agents prevents access for many patients particularly those in developing countries. Given the wide range of treatment options for MG, cost becomes an important factor, and less expensive agents may be considered preferable in many cases. Health economic studies are necessary to understand the cost-effectiveness of novel treatments compared with traditional alternatives. More data is required to develop greater patient and physician confidence in these agents before wide scale use.

## Author Contributions

DM performed the literature search, wrote and edited the manuscript. CB wrote and edited the manuscript. VB planned the review, supervised DM in his review, wrote and edited the manuscript.

## Conflict of Interest

VB has received consultancy fees, honoraria and research support from UCB, Takeda, CSL, Grifols, Alexion, Immunovant, Momenta and Biogen. CB has received consultancy fees from Alexion and UCB. CB has received research support from UCB. The remaining author declares that the research was conducted in the absence of any commercial or financial relationships that could be construed as a potential conflict of interest.
